# Young children's use of derived fact strategies for addition and subtraction

**DOI:** 10.3389/fnhum.2013.00924

**Published:** 2014-01-06

**Authors:** Ann Dowker

**Affiliations:** Experimental Psychology, University of OxfordOxford, UK

**Keywords:** young children, mathematical development, arithmetical reasoning, derived fact strategies, addition, subtraction

## Abstract

Forty-four children between 6;0 and 7;11 took part in a study of derived fact strategy use. They were assigned to addition and subtraction levels on the basis of calculation pretests. They were then given Dowker's (1998) test of derived fact strategies in addition, involving strategies based on the Identity, Commutativity, Addend +1, Addend −1, and addition/subtraction Inverse principles; and test of derived fact strategies in subtraction, involving strategies based on the Identity, Minuend +1, Minuend −1, Subtrahend +1, Subtrahend −1, Complement and addition/subtraction Inverse principles. The exact arithmetic problems given varied according to the child's previously assessed calculation level and were selected to be just a little too difficult for the child to solve unaided. Children were given the answer to a problem and then asked to solve another problem that could be solved quickly by using this answer, together with the principle being assessed. The children also took the WISC Arithmetic subtest. Strategies differed greatly in difficulty, with Identity being the easiest, and the Inverse and Complement principles being most difficult. The Subtrahend +1 and Subtrahend −1 problems often elicited incorrect strategies based on an overextension of the principles of addition to subtraction. It was concluded that children may have difficulty with understanding and applying the relationships between addition and subtraction. Derived fact strategy use was significantly related to both calculation level and to WISC Arithmetic scaled score.

## Introduction

There have been a number of studies of children's use of derived fact strategies in addition and subtraction (Baroody et al., 1983; Russell and Ginsburg, 1984; Beishuizen et al., 1997; Carpenter et al., 1997; Dowker, 1998, 2009; Blote et al., 2000; Star and Rittle-Johnson, 2008; Jordan et al., 2009; Torbeyns et al., 2009; Cowan et al., 2011). Certain derived-fact strategies appear very early (Baroody and Gannon, 1984; Carpenter and Moser, 1984; Siegler and Jenkins, 1989; Cowan and Renton, 1996). One of the earliest is the “counting-on-from-larger” concrete addition strategy, whereby the child adds two numbers (e.g., 2 + 6), by representing the larger number (e.g., with fingers) first, and then “counting-on” the smaller number: “6, 7, 8–it's 8!” This involves implicit use (with or without an explicit knowledge) of the commutativity principle (Baroody and Gannon, 1984; Cowan and Renton, 1996). By contrast, there are many sophisticated strategies involving the use of decomposition and decomposition for multi-digit arithmetic that appear late and appear to characterize unusually skilled mental calculators (Hope and Sherrill, 1987).

There has been rather less research on children's subtraction strategies than on their addition strategies. The use of derived fact strategies might seem even more important with regard to subtraction than addition, since children are generally less able to retrieve subtraction facts than addition facts (Barouillet et al., 2008), so could benefit more from alternative strategies. Yet it may be more difficult for children to use derived fact strategies for subtraction than addition, both because their relative lack of known facts gives them less of a base from which to use them, and because some derived fact strategies for subtraction, such as the “subtraction by addition” strategy (DeSmedt et al., 2010; Peters et al., 2013) depend on some understanding of the inverse relationship between addition and subtraction, which some studies suggest is difficult for children (see below).

Most studies of derived fact strategies have not adjusted the difficulty of the arithmetic problems to the child's arithmetical level, thereby risking on the one hand that some children may find it easier to calculate or retrieve a solution directly than to derive it on the basis of a principle, and on the other hand that they may find the problems so difficult that they refuse to attempt them at all, or make wild guesses. The present study aimed to adapt the problems given to individual children to their previously assessed calculation ability, and to present them with problems just a little too difficult for them to solve unaided. More generally, most studies have not looked at the relationship between derived fact strategy use and arithmetical ability, but have focused more on chronological age differences. The present study looks at relationships between the use of derived fact strategies and both calculation performance level and performance on a standardized arithmetic test emphasizing reasoning. Previous work by this author (Dowker, 1998, 2005, 2009) has focused on individual differences in general readiness to use derived fact strategies (i.e., the total number of such strategies used in a task), whereas this study focuses more on the use of particular strategies.

Thus, the present study investigated 6- and 7–year-olds' ability to use derived fact strategies, based on a range of principles, for both addition and subtraction. The principles were selected for their applicability across a fairly wide range of difficulty. Some derived fact strategies, such as most counting-based strategies or those based on the use of doubles, are mainly applicable to single-digit arithmetic (Carpenter and Moser, 1984; Baroody, 1987); others, including certain decomposition strategies (Beentjes and Jonker, 1987; Beishuizen, 1993; Beishuizen et al., 1997; Carpenter et al., 1997; Fuson et al., 1997) are mainly applicable to multi-digit arithmetic. Important as these strategies are, the present study restricted the strategies under consideration to those that may be used for both single-and multi-digit arithmetic

The *Identity principle*, which is here investigated for both addition and subtraction, is the most basic of arithmetical principles: that if an arithmetical operation produces a given result, then the repetition of the same arithmetical principle will produce the same result. Its use in predicting the result of an arithmetical operation is properly speaking not a “derived-fact strategy” but a “same-fact” strategy. Thus, its inclusion in the study is intended to investigate whether children tend to use the result of one operation to predict the result of another at all, over and above the particular principles that they are able to use in such predictions. This principle has received relatively little attention, but would appear to be a cornerstone of the ability to use derived fact strategies. It was predicted that while the majority of children would use this strategy, a significant number would not.

The *Commutativity principle* is a crucial addition principle, and one which appears to be used with some frequency by primary school children (Baroody et al., 1983; Russell and Ginsburg, 1984; Canobi et al., 1998; Dowker, 1998, 2009; Canobi, 2005). Strategies based on commutativity only hold for addition and therefore are only investigated for that operation.

Simple associativity-based strategies, involving the addition and subtraction of 1, are also investigated. The *N* + *1 principle* for addition is the simplest of the assumptions that result from the broader associativity principle. This is the principle that if one of the addends is increased by 1, then the sum will also be increased by 1. Other related principles, also to be investigated here, include:

The *N* − 1 principle for addition: that if one of the addends is *decreased* by 1, then the sum will also be decreased by 1.
The *Minuend* + *1 principle* for subtraction: that if the minuend is increased by 1, then the remainder will also be increased by 1.
The *Minuend* −*1 principle* for subtraction: that if the minuend is decreased by 1, then the remainder will also be decreased by 1.
The *Subtrahend* +*1 principle* for subtraction: that if the subtrahend is increased by 1, then the remainder will be *decreased by 1*.
The *Subtrahend* −*1 principle* for subtraction: that if the subtrahend is decreased by 1, then the remainder will be *increased by 1*.

Finally, this study investigates strategies based on the inverse relationship between addition and subtraction. Most studies (Bisanz and LeFevre, 1990; Demby, 1993) suggest that strategies based on the *addition/subtraction Inverse principle* (a + b − b = a; if a + b = c, then c − b = a) are among the later-developing derived-fact strategies, and are not typically used until the age of about 10. However, Baroody et al. (1983) found that many 7-and 8-year-olds used this strategy, and that it typically preceded the *N* + 1 strategy. Gilmore and Bryant (2006, 2008) and Robinson and Dubé (2009, 2013) found considerable individual differences in elementary school children's use of this strategy, but some 6-to 9-year-olds pupils used it effectively. A strategy logically related to inversion strategies is the *Complement principle*: if a − b = c, then a − c = b. This has not been much investigated, at least with regard to children in this age range, and will be considered here. It was predicted that neither the addition/subtraction Inverse principle nor the complement principle would be used by a majority of children.

## Methods

### Participants

144 children ranging from 6;0 to 7;11 were tested individually. They came from two state primary schools in Oxford. 79 were boys and 65 were girls. Their mean age was 81.95 months (*SD* = 6.23).

### Procedure

#### Use of principles task

The task was Dowker's (1998, 2009) test of use of arithmetical principles in derived fact strategies. It included tests of strategy use in both addition and subtraction.

#### Addition principles task

In order to evaluate the children's competence in addition calculations, each child was given the mental addition test previously devised to assess children's arithmetical performance prior to an estimation task (Dowker, 1997). It consisted of a list of 20 addition sums graduated in difficulty from 4 + 5, 7 + 1, etc., to 235 + 349. These sums were simultaneously presented orally and visually in a horizontal format. The children's answers were oral.

The sums were as follows:

**Table:** 

(1) 6 + 3	(11) 31 + 57
(2) 4 + 5	(12) 68 + 21
(3) 8 + 2	(13) 52 + 39
(4) 7 + 1	(14) 45 + 28
(5) 4 + 9	(15) 33 + 49
(6) 7 + 5	(16) 26 + 67
(7) 8 + 6	(17) 235 + 142
(8) 9 + 8	(18) 613 + 324
(9) 26 + 72	(19) 523 + 168
(10) 23 + 44	(20) 349 + 234

Testing continued with each child until (s) he had failed to give a correct response to six successive items.

The children were then divided into five levels according to their performance on the mental calculation task. The levels were: Beginning Arithmetic (unable to deal reliably with single-digit addition); Facts to 10 (passed items 1–4 but failed at least 2 of the next 4 items); Facts to 25 (passed items 1–8, but failed at least 2 of the next 4 items); 2-Digit Addition- No Carrying (passed items 1–12, but failed at least 2 of the next 4 items); and 2-Digit Addition –Carrying (passed items 1–16, but failed at least 2 of the final 4 items). Table 1 in the Results section gives the numbers of children at each level, and examples of items that would be within and just outside of their range.

**Table 1:** **Addition strategies used at different levels**.

**Level**	**Beginning arithmetic**	**Facts to 10**	**Facts to 25**	**2-Digit (Carrying)**	**2-Digit (No carrying)**	**Total**		
Problem within range	2 + 2	5 + 3	8 + 6	23 + 44	52 + 39			
Problem just outside range	5 + 3	8 + 6	23 + 44	52 + 39	523 + 168			
*n*	11	34	63	16	20	144		
Mean age in months	79.88 (6.63)	80.98 (6.5)	82.04 (6.3)	83.54 (3.82)	84.65 (5.98)	81.95 (6.23)		
Mean arithmetic scaled score	3.86 (1.07)	8.89 (2.31)	10.62 (3.09)	10.7 (3.68)	12.19 (4.12)	9.97 (3.59)	χ^2^	*p*
Identity	22%	56%	80%	88%	95%	73%	25.66	0.000^**^
Commutativity	9%	38%	65%	82%	70%	57%	28.00	0.000^**^
Addend +1	0%	24%	51%	71%	75%	56%	28.04^**^	0.000^**^
Addend −1	0%	18%	40%	59%	65%	37%	22.06	0.000^**^
Inverse	0%	18%	6%	35%	25%	14%	9.59	0.031^*^

* p < 0.05;

** p < 0.01.

They were then given an arithmetical reasoning test involving *use of arithmetical principles in derived fact strategies*. The technique was used of giving children the answer to a problem and then asking them to solve another problem that could be solved quickly by using this answer, together with the principle under consideration. Problems preceded by answers to numerically unrelated problems were given as controls. The exact arithmetic problems given varied according to the child's previously assessed calculation level of the child, and were selected to be just a little too difficult for the child to solve unaided. Such a set of problems is here termed, as in earlier studies (Dowker, 1998, 2009), the child's *base corresponding set*).

Each child was shown the addition problems, while the experimenter simultaneously read them to him/her. Children were asked to respond orally. The children received three addition problems per principle. The questions about the principles were grouped around the addition problems, so that the children received 6 questions (involving 5 principles and a control question) for one addition problem; then 6 questions for the second addition problem; then 6 questions for the third addition problem.

The principles investigated were as follows:

1. The *Identity principle* (e.g., if one is told that 8 + 6 = 14, then one can automatically give the answer “14,” without calculating, if asked “What is 8 + 6?”).
2. The *Commutativity principle* (e.g., if 9 + 4 = 13, 4 + 9 must also be 13).
3. The *N* ± *1 principle* (e.g., if 23 + 44 = 67, 23 + 45 must be 68).
4. The *N* − *1 principle* (e.g., if 9 + 8 = 17, 9 + 7 must be 17 − 1 or 16).
5. The *addition/subtraction Inverse principle* (e.g., if 46 + 27 = 73, then 73 − 27 must be 46).

For one of the addition problems in each set, the order of presentation of principles was:

Commutativity, Identity, *N* + 1, *N* − 1, Control, Inverse.
For a second problem in each set, the order was:
Inverse, *N* + 1, *N* − 1, Commutativity, Identity, Control.
For the third problem in each set, the order was:
Control, Inverse, *N* − 1, Identity, Commutativity, *N* + 1.
The order of presentation of the addition problems was varied systematically.
Children were allowed 30 s to begin answering a question; if they did not give an answer within that time, the researcher moved on to the next question.

A child was deemed to be able to use a principle if (s) he could explain it and/or used it to derive at least 2 out of 3 unknown arithmetical facts, while being unable to calculate *any* sums of similar difficulty when there was no opportunity to use the principle.

#### Subtraction principles test

The *subtraction principles* part of the Use of Principles Task was also preceded by a calculation pretest, which consisted of a list of 20 subtraction problems, as follows:

**Table:** 

(1) 6 − 2	(11) 68 − 42
(2) 8 − 4	(12) 86 − 44
(3) 10 − 3	(13) 62 − 14
(4) 9 − 5	(14) 43 − 17
(5) 15 − 7	(15) 75 − 38
(6) 13 − 6	(16) 84 − 59
(7) 12 − 4	(17) 326 − 125
(8) 15 − 7	(18) 894 + 513
(9) 37 − 23	(19) 681 − 214
(10) 55 − 32	(20) 572 − 348

The children were then divided into four levels according to their performance on the mental calculation task. The levels were: Beginning Arithmetic (unable to deal reliably with single-digit subtraction); Facts to 10 (passed items 1–4 but failed at least 2 of the next 4 items); Facts to 25 (passed items 1–8, but failed at least 2 of the next 4 items); and 2-Digit Subtraction (passed items 1–12, but failed at least 2 of items 13–16 and/or of items 17–20). Originally, the 2-Digit Subtraction group was divided into two groups, as with addition: 2-Digit-No Borrowing, and 2-Digit- Borrowing. However, as only 8 children would have met criteria for the 2-Digit-Borrowing group, they were grouped together, for the purposes of the present study, with those who could only carry out 2-digit subtraction when borrowing was not involved. Table 3 in the Results section gives the numbers of children at each level, and examples of items that would be within and just outside of their range.

The questions about the principles were grouped around the subtraction problems, so that the children received 8 questions (involving 7 principles and a control question) for one addition problem; then 8 questions for the second addition problem; then 8 questions for the third addition problem

The principles investigated for *subtraction* were as follows, in order of their difficulty for the children:

1. The *Identity principle* (e.g., if one is told that 12 − 5 = 7, then one can automatically give the answer “7,” without calculating, if asked “What is 12 − 5?”).
2. The *Minuend* ±*1 principle* (e.g., if 67 − 45 = 22, 68 − 45 must be 23).
3. The *Minuend* −*1 principle* (e.g., if 572 − 348 = 224, 571 − 348 must be 223).
4. The *Subtrahend* ±*1 principle* (e.g., if 9 − 6 = 3, 9 − 7 must be 2).
5. The *Subtrahend* −*1 principle* (e.g., if 37 − 23 = 14, 37 − 22 must be 15).
6. The *addition/subtraction Inverse principle* (e.g., if 681 − 214 = 467, then 214 + 467 must be 681.
7. The *Complement* principle (e.g., if 11 − 3 = 8, 11 − 8 must be 3).

For one of the subtraction problems in each set, the order of presentation of principles was:

Complement, Minuend + 1, Subtrahend + 1, Inverse, Minuend −1, Subtrahend −1, Identity, Control.
For a second problem in each set, the order was:
Minuend−1, Subtrahend +1, Minuend +1, Inverse, Identity, Minuend −1, Control, Complement.
For the third problem in each set, the order was:
Control, Identity, Subtrahend +1, Minuend +1, Subtrahend −1, Complement, Minuend −1, Inverse.
The order of presentation of the subtraction problems was randomly varied.
Children were allowed 30 s to begin answering a question; if they did not give an answer within that time, the researcher moved on to the next question.
The order of presentation of addition and subtraction was randomly varied.
In addition, the children were given the Arithmetic subtest of the Wechsler Intelligence Scale for Children or WISC (Wechsler, 1991).

## Results

As no children calculated the answers to the control questions within the time given, responses to control questions will not be analyzed here.

### Results for addition

Table 1 gives the percentage of responses at each level using each principle in derived fact strategies for addition.

Chi-square tests were carried out to investigate whether there were significant differences between the different levels as regards the frequency of each strategy. The chi-square value and *p* value are given in the final two columns of Table 1. In all the chi-square comparisons, there were 4° of freedom.

*Post-hoc* tests were then carried out to investigate which group differences were causing the significant effects. For the Identity principle and the Addend −1 principle, the significant differences were between Beginning Arithmetic and each of the other levels except for the Facts to 10 level; and between the Facts to 10 level and the 2-Digit (Carrying) level. For the Commutativity principle and the Addend +1 principle, the significant differences were between Beginning Arithmetic and each of the other levels except for the Facts to 10 levels; and between the Facts to 10 level and both the 2-Digit (No Carrying) the 2-Digit (Carrying) levels. For the Inverse principle, there was a borderline significant difference between the Beginning Arithmetic and the 2-Digit (No Carrying) level, and no other group differences reached significance.

Entry method nominal logistic regressions were carried out with each principle (Used or Did Not Use) as the dependent variable. The covariates were Age in months and WISC Arithmetic (Scaled Score). The chi-square and p-values for these regressions are given in Table 2.

**Table 2:** **Results of nominal logistic regressions on the use of addition strategies with age and arithmetic scaled score as covariates**.

**Principle used**	**Age in months: χ^2^**	**Age in months: *p***	**Arithmetic scaled score: χ^2^**	**Arithmetic: scaled score: *p***
Identity	4.505	0.034^*^	4.92	0.034^*^
Commutativity	4.66	0.031^*^	3.885	0.049^*^
Addend +1	2.73	0.099	8.045	0.005^**^
Addend −1	3.32	0.069	7.64	0.006^**^
Inverse	1.43	0.232	0.027	0.87

* p < 0.05;

** p < 0.01.

In all chi-square comparisons, df = 1.

### Results for subtraction

Table 3 gives the percentage of responses at each level using each principle in derived fact strategies for subtraction. With regard to the Subtrahend +1 and Subtrahend −1 strategies, two percentages are given. The first percentage given is that for use of a common but incorrect strategy: that of assuming that if a − b = c, then a − (b + 1) = c + 1 (instead of c − 1), or that if a − b = c, then a − (b − 1) = c − 1 (instead of c + 1). The second percentage is for the use of the correct strategy.

**Table 3:** **Subtraction strategies used at different levels**.

**Level**	**Beginning arithmetic**	**Facts to 10**	**Facts to 25**	**2-Digit subtraction**	**Total**		
Problem within range	?	6–3	12–5	58–34			
Problem just outside range	6–3	12–5	58–34	82–26			
*n*	18	56	48	22	144		
Mean age in months	79.88 (6.63)	80.98 (6.5)	82.87 (5.84)	85.63 (4.59)	81.95 (6.23)		
Mean arithmetic scaled score	4.82 (1.94)	9.39 (2.58)	11.4 (3.33)	12.31 (3.61)	9.97 (3.59)	**χ^2^**	*p*
Identity	17%	61%	77%	86%	65%	29.49	0.000^**^
Minuend +1	0%	23%	54%	71%	38%	35.26	0.000^**^
Minuend −1	0%	21%	50%	71%	56%	9.42	0.022^*^
Subtrahend +1	0% + 6%	20% + 4%	60% + 4%	55% + 22%	38% + 6%	1.92^a^ 9.66^b^	0.775^a^ 0.02^*^^b^
Subtrahend -1	0% + 6%	20% + 2%	54% + 6%	43% + 29%	33% + 7%	2.45^a^ 11.23^b^	0.57^a^ 0.009^**^^b^
Complement	0%	18%	6%	35%	14%	9.43	0.022^*^
Inverse	0%	7%	17%	27%	12%	8.56	0.026^*^

* p < 0.05

** p < 0.01

^a^Analysis for correct strategy only.

^b^Analysis for combination of correct strategy with common incorrect strategy.

Chi-square tests were carried out to investigate whether there were significant differences between the different levels as regards the frequency of each strategy. The chi-square value and *p* value are given in the final two columns of Table 3. In the case of the Minuend +1 and Minuend −1 strategies, two comparisons were made: one taking only the correct strategy into account; and one combining use of the correct strategy and the common incorrect strategy. In all the chi-square comparisons, there were 3° of freedom.

*Post-hoc* tests were then carried out to investigate which group differences were causing the significant effects. For the Identity principle, the significant differences were between Beginning Arithmetic and each of the other levels. For the Subtrahend +1 principle and the Subtrahend −1 principle, the significant differences were between Beginning Arithmetic and each of the other levels; and between the Facts to 10 level and each of the other levels. Two different *post-hoc* analyses were carried out for the Subtrahend +1 and Subtrahend −1 principles: for the correct strategy alone, and for the correct strategy combined with the common incorrect strategy. For the correct strategy alone, no group differences were significant for these principles. For the combination of the correct and the common incorrect strategy, the significant differences, in the case of both the principles, were between the 2-Digit Subtraction level and every other level. For the Complement principle and the Inverse principle, there were significant differences between the Beginning Arithmetic and the 2-Digit Subtraction level, and no other group differences reached significance.

Entry method nominal logistic regressions were carried out with each principle as the dependent variable. The dependent variable was binary (Used vs. Did Not Use). In the case of the Minuend +1 and Minuend −1 principles, two different analyses were done: (a) for Correct Strategy Use alone and (b) for Correct Strategy Use combined with Common Incorrect Strategy Use. The covariates were Age in months and WISC Arithmetic (Scaled Score). The chi-square and p-values for these regressions are given in Table 4.

**Table 4:** **Results of nominal logistic regression on use of subtraction strategies with age and arithmetic scaled score as covariates**.

	**Age in months: χ^2^**	**Age in months: *p***	**Arithmetic scaled score: χ^2^**	**Arithmetic: scaled score: *p***
Identity	4.86	0.041*	6.84	0.009^**^
Minuend +1	8.265	0.004^**^	14.77	0.000^**^
Minuend −1	3.3	0.068	10.9	0.001^**^
Subtrahend +1	1.86^a^; 3.3^b^	0.24^a^; 0.07^b^	0.12^a^; 1.37^b^	0.73^a^; 0.24^b^
Subtrahend −1	4.42^a^; 4.43^b^	0.035^*^^a^; 0.035^*^^b^	0.57^a^; 0.7^b^	0.45^a^; 0.4^b^
Complement	4.915	0.027^*^	2.13	0.145
Inverse	0.88	0.348	5.45	0.02^*^

* p < 0.05;

** p < 0.01.

^a^Analysis for correct strategy only.

^b^Analysis for combination of correct strategy with common incorrect strategy.

### Children's justification of their answers

Most (91%) of children classed as using the principles were able to justify their answers.

Typical justifications included:

(Identity); “It's the same!”
(Commutativity): “Those numbers are just the same, but the other way round.”
(*N* + 1 principle for addition; Minuend +1 principle for subtraction): “It's just one more.”
(*N* − 1 principle for subtraction; Minuend −1 principle for subtraction): “It's just one less.”
(Subtrahend +1 principle for subtraction): (Usually, incorrectly): “It's just one more.” (Correctly): “That's one more, so the answer has to be one less.”
(Subtrahend −1 principle for subtraction): (Usually, incorrectly): “It's just one less.” (Correctly): “That's one less, so the answer has to be one more.”
(× 10) principle: “You just add on a 0.”
(Inverse principle): “Because that (a + b) = c, so c take away that (a) must be (b).” (Of course the child used the actual numbers rather than letters.)
(Complement principle): “If that (a) take away that (b) = c, then that (a) take away that (c) must be b.”

## Discussion

This study shows that many 6- and 7-year-olds children can make explicit use of derived fact strategies in addition and subtraction. There is, however, a great deal of variation in the use of such strategies in this age range, influenced by both by the particular strategies involved, and by children's calculation ability.

### Use of particular strategies

The most basic principle, Identity, was used with by far the greatest frequency; and is the only strategy that was used more than once or twice at the Beginning Arithmetic level. It was still only used by a minority of children at this level, however; and was not used universally even at the higher levels. This was followed by commutativity of addition, supporting other studies that suggest that this principle is used earlier than most other arithmetical principles (Baroody et al., 1983; Cowan and Renton, 1996; Canobi et al., 1998, 2003).

The strategy of using commutativity is followed in frequency by strategies that involve adding, or (to a lesser extent) subtracting, 1 from a problem component and thereby to the result.

Strategies of the latter type could be, and often were, used incorrectly as well as correctly. When used for addition, they tended to be used correctly; but this was not the case for subtraction, where the Subtrahend +1 and Subtrahend −1 problems were more likely to lead to incorrect than correct strategy use. Children are more likely, if told that a − b = c, to deduce that a − (b + 1) = c + 1, than correctly that a − (b + 1) = c − 1. In other words, when using this class of strategies, they often fail to make appropriate use of compensation. This may in part reflect procedural difficulties, perhaps relating to working memory limitations. However, when considered in conjunction with the children's common failure to use the addition-subtraction inverse principle for addition or subtraction, or the complement principle for subtraction, it probably also reflects a difficulty in understanding the relationships between addition and subtraction. The arithmetical relationships most accessible to children appear to be those appropriate to addition, and these are sometimes inappropriately extended to subtraction. It may be that the same is true of relationships between addition and other arithmetical operations; e.g., MacCuish (1986) found that 9- and 10-year-olds children overextended certain addition principles to multiplication.

With regard to strategies involving use of the inverse relationship between addition and subtraction, results of the present study are far more consistent with those of Bisanz and LeFevre (1990) than with those of Baroody et al. (1983), in that strategies of this nature were used very infrequently. The logically related complement strategy for subtraction was used even more rarely.

This is particularly striking, since these children were being taught mathematics according to the National Numeracy Strategy (DfEE, 1999), which explicitly recommended teaching children to understand the inverse relationship between addition and subtraction from the second year of primary school onwards. Nevertheless, the principle was only used by about one in ten children, similar to findings for a sample studied before the explicit introduction of this concept into the English school curriculum (Dowker, 1998). This suggests that children, at least under the age of 8, do not readily make use of this principle in arithmetic.

However, this may not be the case for *all* arithmetical tasks. Gilmore and Bryant (2006, 2008) found that 6-to 9-year-olds children did often make use of derived fact strategies involving inverse relationships between addition and subtraction. They performed better and more accurately on such problems as “15 + 12 − 12=□” than on control problems such 11 + 11 − 7 = □.” An explanation for the discrepancy in results might be that children are better at noticing and making use of relationships between addition and subtraction *within* an arithmetic problem than *between* two arithmetic problems. If there is an addition and a subtraction within the *same* problem it is perhaps harder to treat them as unrelated—“one's adding and one's taking away”—than if the task involves perceiving and using a relationship between an addition problem and a subtraction problem. This provides further evidence that the ability to use derived fact strategies is not “all or nothing” and may be highly dependent on context and mode of presentation of a task.

### Do success and failure in the derived fact strategy test always reflect use of principles?

So far in this paper, “use of principles” and “use of derived fact strategies” have been discussed almost as though they were synonymous; but of course the relationship between the two is likely to be far more complex. With all of the arithmetical principles discussed here, there are two separate issues: whether a child understands an arithmetical concept or principle, and whether they use this principle appropriately in an arithmetical strategy. Some principles may not be used in derived fact strategies because the children have no access to the principles. On the other hand, children may understand an arithmetical principle or relationship, but not apply it appropriately.

The present study involved explicit use of derived fact strategies in a task involving arithmetic problems presented in symbolic format, and not embedded in a practical or social context. Some studies have suggested that children may be more likely to use derived fact strategies when problems are presented in concrete form (Bryant et al., 1999) or if the task requires only implicit rather than explicit use of the principle (Siegler and Stern, 1998). Canobi et al. (1998) studied 6-to 8-year-olds' use of derived fact strategies based on commutativity and associativity, and their evaluations of puppets using these strategies. They were considerably better at judging and justifying the appropriateness of a puppet's use of such strategies than at using the strategies themselves. It is therefore likely that the present study gives a somewhat conservative estimate of the extent of derived fact strategy use in young children.

However, studies also suggest that elicited use of derived fact strategies is not the *most* difficult task. Children and even adults tend to be better at using derived fact strategies appropriately when these are instructed or directly elicited, than at using them spontaneously, though there is a strong correlation between elicited and spontaneous use of such strategies (Gaschler et al., 2013).

A review by Prather and Alibali (2009) indicates that context and mode of assessment may have a significant impact on whether children use such strategies. Moreover, it is possible that children may sometimes have failed to use a strategy because of a coincidental procedural error or momentary distraction, rather than because of a failure to understand the principle. The fact that the criterion for success on a principle was use of the relevant strategy for two out of three arithmetic problems (rather than all three) reduces this risk, but does not eliminate it completely.

The question also arises of whether the reverse may have happened at times: could children have responded correctly to some items because they calculated from scratch and did so accurately, rather than because they used the principle? However, while this possibility can never be totally ruled out, it is unlikely to have occurred in most cases because (1) the sets of problems given to individual children were selected on the basis of the pretest indicating that they would be too difficult for them to calculate mentally; (2) they were not able to calculate the control problems mentally; (3) in the vast majority of cases, they were able to justify their correct answers.

### Relationships between derived fact strategies and arithmetical ability

Although discrepancies can and do occur, in both directions, between calculation performance level and extent of derived fact strategy use (Dowker, 1998, 2009), the two are very strongly associated (see Tables 1, 3). This was true despite the fact that the difficulty of the arithmetic problems given was adjusted according to the children's calculation performance levels. Only a minority of children at the Beginning Arithmetic levels for addition and subtraction used any derived fact strategies. The use of such strategies became more frequent at the Facts to 10 levels, and increased sharply as children reached the Facts to 25 level and beyond. This increase with calculation performance level was found for both addition and subtraction; and was significant for all strategies except for the Complement principle for subtraction, perhaps due to floor effects for this principle.

Scaled score on an arithmetical reasoning task was also a strong predictor of most strategies, showing a significant relationship to use of all strategies except some of the more difficult ones: the Inverse principle in the addition task; and the Subtrahend +1, Subtrahend −1 and Complement principles in the Subtraction task. Thus, the use of most derived fact strategies is closely related to arithmetical ability. The relationship to chronological age is less strong, but is present for Identity and Commutativity in addition and for Identity, Minuend +1 and Subtrahend −1 in subtraction. These children were all within a relatively limited age range (6;0 to 7;11) and age might be found to have a stronger influence in a group with a wider age range.

The relationships that were found between derived fact strategy use and both calculation performance levels and WISC Arithmetic could indicate that a certain level of arithmetical knowledge is a prerequisite for the use of such strategies. Alternatively, the derived fact strategies may develop first, and contribute to an improvement in calculation performance.

As pointed out by Dowker (2009), these alternative possibilities have some parallels with the “some principles first” and “skills first” theories of the relationship between counting principles and procedures. Findings (Dowker, 2008) with regard to the existence of both a strong correlation and the existence of discrepancies in both directions in individual children suggest some degree of “mutual development” or iterative relationship between the two (Baroody and Ginsburg, 1986; Cowan et al., 1996). Rittle-Johnson et al. (2001) have suggested that this extends to the iterative development of principles and skills in the later development of arithmetic. This would be consistent with the results of this study, showing a strong relationship between derived fact strategy use and arithmetical ability as measured both by addition and subtraction performance levels and by the WISC Arithmetic test, but at the same time, showing discrepant performance (e.g., Tables 1, 3 show that some children at the Facts to 10 level used the addition/subtraction inverse strategy, and some at the higher levels failed to use Identity—though the latter was rare, and might possibly be explainable on the basis of momentary distraction or procedural error).

Dowker (2009) found the relationships between derived fact strategy use and performance on standardized arithmetic tests to be less strong in children with mathematical difficulties than in other children, which may indicate that the iterative integrative process occurs less effectively in this group than among typically achieving children.

Further research is needed to investigate the extent to which both age and level of mathematical achievement may influence the relationships between calculation and derived fact strategy use. Certainly, the evidence suggests that there are children, both among low and typical achievers in mathematics, whose derived fact strategy use is considerably better than would be expected from their calculation ability (Dowker, 1998; Gilmore and Bryant, 2006, 2008). Further studies of the characteristics of such children might give us a greater understanding of the levels of functional independence and interdependence between derived fact strategy use and other arithmetical abilities.

### Other areas for further research

Much more research, and in particular longitudinal research, is needed if we are to fully understand the nature, foundations and development of derived fact strategies. This must involve research into the order in which such strategies develop, and whether any particular strategies are prerequisites for any other strategies. It must also involve studying the nature and direction of predictive relationships between derived fact strategies, calculation performance, and arithmetical concepts; and, in particular, whether derived fact strategies are more dependent on principled knowledge or the ability to implement strategies in arithmetic. Intervention studies would be crucial here: would training in derived fact strategies lead to improvement in calculation, and/or vice versa?

With regard to this issue, it is also important to investigate the effects of context and task presentation mode on performance. Research should also go beyond arithmetic in examining whether any domain-general abilities have a specific role to play in the development and use of derived fact strategies.

Moreover, it would be desirable to investigate the neural mechanisms involved in understanding and using derived fact strategies. Studies of patients have indicated that double dissociations can occur between retrieval and derived fact strategy use (Warrington, 1982; Delazer, 2003). Now that it is increasingly feasible to carry out brain imaging studies with children, researchers should investigate whether the network of brain areas involved in the use of derived fact strategies differs in any way from that involved in other aspects of arithmetic, and whether this changes with development.

### Conflict of interest statement

The author declares that the research was conducted in the absence of any commercial or financial relationships that could be construed as a potential conflict of interest.
